# 2461 Increasing butyrate levels by microbial manipulation or drug administration to delay Parkinson’s disease progression

**DOI:** 10.1017/cts.2018.86

**Published:** 2018-11-21

**Authors:** Stephanie M. Garcia, Wenbo Zhou, Curt R. Freed

**Affiliations:** 1 University of Colorado at Denver; 2 School of Medicine, University of Colorado, Denver

## Abstract

OBJECTIVES/SPECIFIC AIMS: Determine if synthetic or endogenously produced butyrate can delay Parkinson’s disease (PD) progression, attenuate PD associated GI dysfunction, and impact the gut-microbiota in mice expressing human mutant aSyn. METHODS/STUDY POPULATION: Two transgenic mouse models expressing human mutant alpha-synuclein (aSyn) will be used. Transgenic mice expressing aSyn A53T display GI dysfunction before motor deficit onset and will be used to investigate treatment impact on PD associated GI dysfunction. Mice expressing aSyn Y39C more accurately recapitulate age-related neuropathology and behavioral deficits and will be used to assess treatment impact on PD-associated neuropathology, motor, and cognitive function. Mice will receive a synthetic sodium butyrate, sodium phenylbutyrate, or a synbiotic treatment regimen for 3 months. Disease progression will be assessed by aSyn brain and gut neuropathology, brain and gut inflammatory status, behavioral deficits, and gastrointestinal function. In addition, fecal and gut-microbiota composition and neuroprotective gene expression in the brain will be investigated. RESULTS/ANTICIPATED RESULTS: Our preliminary data shows that both sodium butyrate and sodium phenylbutyrate delay disease progression in aSyn Y39C mice. Butyrate-treated mice have reduced aSyn oligomerization, reduced Lewy body formation, and improved motor and cognitive function compared to placebo-treated mice. 16S rRNA sequencing did not reveal fecal-microbiota shifts between treatment groups or with age progression. Further analysis assessing expression levels for genes with anti-oxidant and protein degradation roles will be performed to determine if sodium butyrate and sodium phenylbutyrate similarly impact cellular mechanisms to delay neurodegeneration. Our future experiments will focus on comparing sodium butyrate and synbiotic treatment outcomes in aSyn A53T mice. DISCUSSION/SIGNIFICANCE OF IMPACT: Our lab developed a Tg mouse model that more accurately recapitulate age-related symptoms, pathology, and mechanisms observed in PD patients compared with animal models onset by neurotoxins. Our use of an age-dependent model of a severe form of Parkinsonism, DLB, will better predict clinical outcomes in PD populations. We will be the first to assess if elevating select microbial product production enhances neuroprotective brain activity in a PD model. Results obtained will further characterize gut-brain axis communication mechanisms. These proposed experiments will be the first to determine if elevating microbial products improves GI deficits associated with PD and may lead to insight on the gut-brain axis role in PD. Overall, this proposal will be the first to investigate a novel, highly accessible treatment with the potential to delay PD progression and target motor, cognitive, and GI deficits associated with PD. Due to the current FDA approval of probiotics and prebiotics that enhance butyrate production, results obtained may be quickly translated for clinical use.

